# A Simple and Fast Method to Synthesize Cubic Iridium Nanoparticles with Clean Surface Free from Surfactants

**DOI:** 10.3390/nano9010076

**Published:** 2019-01-08

**Authors:** Rongrong Zhang, Xuan Liu, Litong Shi, Xin Jin, Yanchao Dong, Kang Li, Xihui Zhao, Qun Li, Yujia Deng

**Affiliations:** School of Chemistry and Chemical Engineering, Qingdao University, Qingdao 266071, China; zhangrr0809@163.com (R.Z.); liuxuan2795@163.com (X.L.); 15666191885@163.com (L.S.); 17863966345@163.com (X.J.); 17805420783@163.com (Y.D.); lk17866634806@163.com (K.L.); zhaoxihui@qdu.edu.cn (X.Z.)

**Keywords:** Iridium nanoparticles, cubic, Cu foil, free from surfactants, galvanic reaction

## Abstract

Cubic Iridium nanoparticles without any surfactants on the surface have been synthesized successfully in this work. The process of synthesis was quite simple by just injecting one drop of 400 µL solution containing Iridium precursor onto Cu foil (1 cm × 1 cm), and through galvanic reaction between the Ir precursor and Cu foil, the cubic Iridium nanoparticle could be obtained quite quickly (<30 s). The Cu foil played the roles of both reducing agent and substrate. This method could also be employed to synthesize cubic nanoparticles of other Pt-group metals such as Rh. By employing this method, cubic metal nanoparticles with surfactant-free surfaces could be produced economically and efficiently, and as a result, a realistic relationship between structure and catalytic activity could be established.

## 1. Introduction

In recent decades, nanoparticles have boosted the development of catalysis, due to their high surface-area-to-volume ratio resulting in increased exposure of the catalytic active sites, and reduced usage of noble metals [1,2,3]. Nanocatalysts produced from Pt-group metals have been widely used in energy fields such as fuel cells [4,5]. Fundamental studies indicated that the surface structure could have significant effects on their catalytic performances. For example, Pt (110) could have higher catalytic activity than that of Pt (111) towards oxygen reduction reaction in HClO_4_ solution and similar phenomena have been also found in Ir catalytic processes [1,6,7,8]. To enhance the catalytic performances of Pt-group metals for certain reaction, nanoparticles enclosed with specified surface structure are preferred, and this can be achieved when shape-controlled synthesis of the nanomaterials is used. Recent decades have witnessed the quick development of shape-controlled synthesis of nanomaterials. Shape-controlled synthesis of Pt-group metal nanocrystals (NCs), such as Pt, Pd, were of great success and the synthesized nanomaterials have shown excellent catalytic activity [4,9,10]. Among Pt-group metals, Ir was supposed to be an ideal catalyst candidate for oxygen evolution reaction (OER) due to its activity and stability [8,11,12]. However, the realization of shape-controlled synthesis of pure Ir nanocrystals remained a significant challenge. Xia employed cubic Pd nanocrystals as seed and synthesized cubic Pd-Ir core-shell nanoparticles, but the process was a little complex and the conditions were critical at high temperature [13]. In addition, most reported synthesis processes of shaped nanoparticles of Pt-group metals employed surfactants such as PVP, CTAB, etc. [10,14]. These surfactants could adsorb strongly on the surface of the nanoparticles thus affecting its catalytic activity. It is widely accepted that a better understanding of the correlation between surface structure and catalytic performance can be obtained when the surface of the metal is free of adsorbed contaminants [15]. In this respect, the development of an effective method to synthesize nanoparticles without surfactants on the surfaces became an extremely important prerequisite for subsequently evaluating their catalytic performances. So far, despite much effort, there has been limited success in finding efficient methods for the removal of adsorbed contaminants from the surface of nanocatalysts [16,17,18,19]. Since it is difficult to remove the surfacants after they are adsorbed on the surface of nanocatalysts as synthesized, a simple strategy would be to avoid introducing them at the stage of synthesis. Although shaped Pt, Pd nanoparticles with clean surfaces free from surfactants have been acquired by this strategy [20,21,22], no shaped Iridium nanoparticles have been similarly produced. Therefore, shape-controlled synthesis of Ir nanoparticles with clean surfaces could be quite appealing. Hitherto, Kibler et al. employed square-wave potential procedure and successfully electrodeposited Ir nanoparticles on a glassy carbon electrode. Although the nanoparticles were free from surfactants, they were nanospheres enclosed with poly-crystal facets [23]. In earlier work, Dai et al. reported that Pt nanocubes could be directly deposited onto Cu foil by reacting appropriate aqueous solutions of K_2_PtCl_4_ with Cu foil via a galvanic displacement reaction for given reaction times [22]. The method has been successfully used to synthesize Pt and Au cubic nanoparticles without surfactants. The finding from their study suggests that it is possible to synthesize cubic Iridium nanoparticles with clean surfaces. Herein, we report a simple method for the synthesis of surfactant-free cubic Ir nanoparticles that can be extended to the production of cubic nanoparticles of other noble metals, such as rhodium. 

## 2. Experimental

IrCl_3_ (GR) and Na_3_RhCl_6_ (GR) were purchased from Rhawn and Alfa Aesar, respectively. Before reaction, Cu foil (1 cm × 1 cm) was pretreated by putting it in sulfuric acid for 5 s to clean the surface and then was washed by Milli-Q water (18.2 MΩ cm) to remove the acid. All the solution used was prepared with Milli-Q water. In a typical process of synthesis, Ir nanoparticles were produced by injecting a drop of 400 µL of 2 mM IrCl_3_ + 10 mM CuCl_2_ aqueous solution onto the pretreated Cu foils by pipette at room temperature for a certain period of time, followed by thoroughly rinsing with Milli-Q water. For the synthesis of Rh nanoparticles, the process was similar but with a drop of 400 µL of 2 mM Na_3_RhCl_6_ + 10 mM CuCl_2_ aqueous solution. For the oxidation of 3,3′,5,5′-tetramethylbenzidine (TMB) by H_2_O_2_, the reactions were carried out in 5 mL potassium biphthalate buffer solution (pH 4.0), containing 2 M H_2_O_2_ and 0.8 mM TMB as the substrates and the Cu foil with Iridium nanoparticles was put in the solution as catalysts. The morphology and structure of the nanoparticles were characterized by scanning electron microscopy (SEM, Hitachi S-4800), transmission electron microscopy (TEM, JEM-2100 at 200 kV). An energy-dispersive X-ray spectroscopic (EDS) detecting unit was used for the element analysis. UV-vis spectra were obtained on a Shimadzu UV3150. X-ray diffraction (XRD) measurements were performed on X-ray diffractometer (D/MAX-RB) using CuKα radiation (λ = 0.15418 nm).

## 3. Results and Discussion

**Figure 1:**
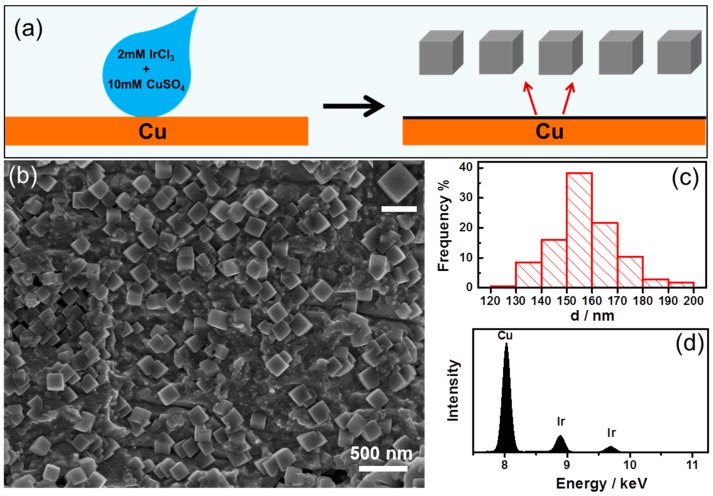
(**a**) Schematic illustration of metal nanoparticle deposition on Cu foil. (**b**) Overview SEM image of the Iridium nanoparticles and the inset was high magnification, and the scale bar in it is 200 nm. (**c**) Size distribution of the synthesized Iridium nanoparticles. (**d**) EDS of the Iridium nanoparticles as synthesized.

Figure 1 shows the SEM image of Ir nanoparticles obtained by galvanic displacement reaction between Cu foils and aqueous IrCl_3_ solutions for a constant deposition time of 60 min. Figure 1a shows the schematic illustration of metal nanoparticle deposited on Cu foil through galvanic process. A drop of 400 µL of 2 mM IrCl_3_ + 10 mM CuCl_2_ aqueous solution was injected onto the pretreated Cu foils by pipette and after reaction for 60 min, the Ir nanoparticles formed on the surface. Figure 1b displays a low-magnification SEM image of the as-prepared Ir nanoparticles. It has found that the Ir NCs with cubic shape were the dominant products with a typical yield of nearly 100%. The nanoparticles distributed evenly on the surface of Cu foil. The high-magnification SEM image in the inset in Figure 1a illustrates clearly the perfect cubic shape of the Ir NCs, with sharply faceted edges. Figure 1c shows the size distribution of the Ir nanoparticles as synthesized. The size distribution of nanoparticles was determined by measuring the edge of each nanoparticle. As shown in Figure 1c, the size of the cubic Ir NCs varies from 120 to 200 nm with an average of 157.7 nm, relative standard deviation (RSD) = 7.7%. Figure 1d shows the EDS of the sample and it indicated that the nanoparticles grown on the Cu foil were Iridium. These results indicate that the cubic Iridium nanoparticles were successfully synthesized by the simple galvanic reaction. 

**Figure 2:**
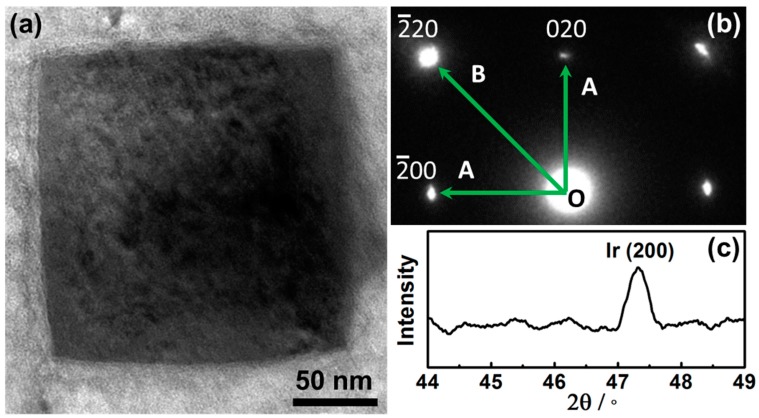
(**a**) TEM of a typical Ir nanoparticle. (**b**) SAED of the nanoparticle. (**c**) XRD of the nanoparticles as synthesized on Cu foil.

Figure 2a shows the TEM image of a single Ir nanoparticle alone [001], clearly demonstrating the cubic shape. This was fully consistent with the SEM observations (Figure 1b). Figure 2b shows the selected-area electron diffraction (SAED) pattern of the Iridium nanoparticle and the fourfold symmetry of the SAED pattern confirmed that the cubic Ir NC was a single crystalline. The angle between OA and OB was 45°, and the distance ratio between OB and OA is 1.414, the results indicated that the formed cubic Ir nanoparticles were enclosed by (100) facets. Figure 2c shows the XRD of the nanoparticles as shown in Figure 1 and the peak at 47.3° confirmed the existence of the (200) diffraction peak for cubic Ir (JCPDS 06-0598) [24]. The results further indicated the cubic Iridium nanoparticles were enclosed with (100) facets.

**Figure 3:**
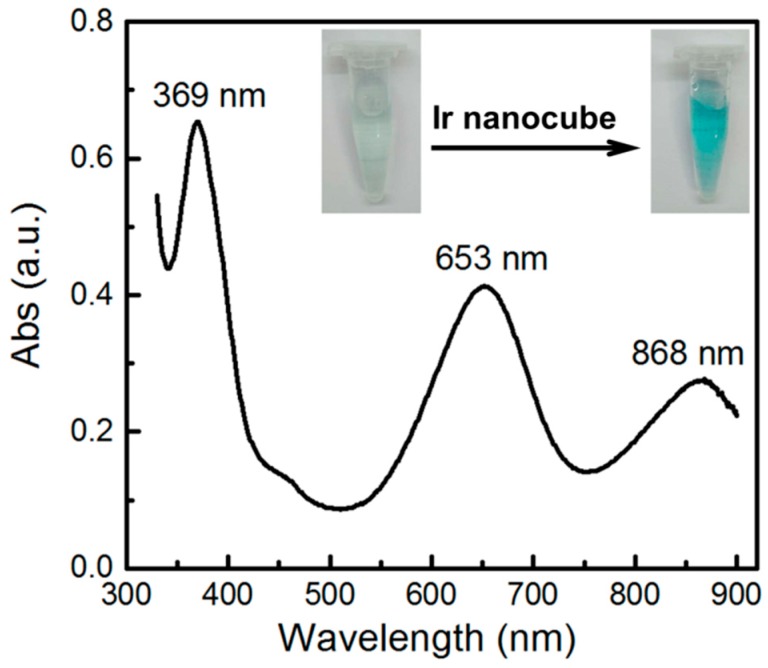
UV-vis spectrum characterization of TMB oxidation by H_2_O_2_ catalyzed by cubic Ir nanoparticles.

It was reported that Ir nanoparticles could catalyze the oxidation of 3,3′,5,5′-tetramethylbenzidine (TMB) by H_2_O_2_ and yielding a blue-colored product with a maximum absorbance at 653 nm [13]. We employed this method to further characterize the as-synthesized Ir cubes. As shown in Figure 3, In the presence of the cubic Ir nanoparticles, the oxidation of TMB by H_2_O_2_ could be achieved and yielding a blue-colored product with a maximum absorbance at 653 nm and the color change was quite obvious from colorless to blue as the catalysis reaction proceeded, which was consistent well with that reported in literature. This result further confirmed that the nanoparticles were Iridium.

**Figure 4:**
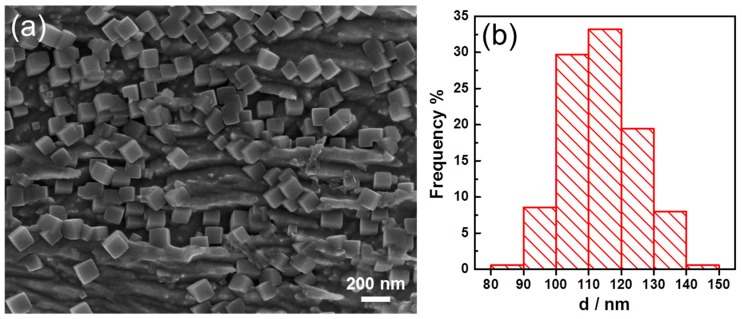
(**a**) SEM of the Iridium nanoparticles synthesized for 30 s. (**b**) Size distribution of the synthesized Iridium nanoparticles.

When we injected the drop of solution onto the Cu foil, we found that the surface of the Cu foil turned black immediately. This made us interested to check how fast the cubic Ir nanoparticles could form. To investigate this, we conducted the experiment as before but made the time much shorter until it was only 30 s. In addition, the results are shown in Figure 4. In Figure 4a, it can be seen that on the surface of the Cu plate, the nanoparticles were distributed evenly on the surface and most of them were cubic. In Figure 4b, it can be seen that most of the size ranged from 80 to 150 nm, and the average size was 114.2 nm, and RSD was 9.5%. By checking carefully, we found there were several nanoparticles much smaller than the average size, but these nanoparticles could be seen clearly with well cubic shape. The results demonstrated that the galvanic displacement reaction could be quite fast, and the cubic shape should be achieved quickly (<30 s) and grew to a large size soon. Since only one drop of 400 µL of solution containing Iridium precursor was used to react with the Cu foil and the galvanic reaction was fast, the nanoparticles did not grow so much from 30 s to 60 min as the average size changed from 114.2 to 157.7 nm. This meant that we could employ this method to obtain cubic Iridium nanoparticle in short time with limited reactants and this could facilitate to produce them as catalysts at large scale. 

**Figure 5:**
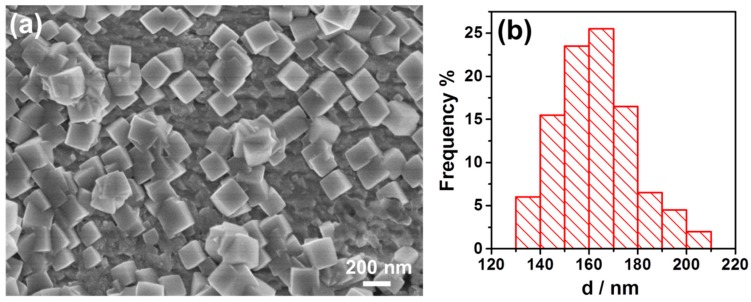
(**a**) SEM of the Rh nanoparticles synthesized for 3min. (**b**) Size distribution of the Rh nanoparticles as synthesized.

Compared with the method described by Dai to synthesize cubic Pt nanoparticles, we have simplified the synthesis process with only one drop of precursor solution. Thus, each drop of the solution could be a reaction spot. This made the synthesis more economic and efficient. This simple method could be expanded to synthesize other cubic nanoparticles of Pt-group metals such as Rh. Figure 5 shows the Rh nanoparticles synthesized by the same method but with a drop of solution containing Rh precursor for 3 min. In Figure 5a, most of the nanoparticles were cubic shape, and though some of the nanoparticles grew together, their cubic profiles were clear. Figure 5b shows size distribution of the Rh nanoparticles as synthesized. It shows the size of nanoparticles ranged from 130 to 210 nm and the average size was 163.1 nm and RSD was 9.6%.

## 4. Conclusions

In conclusion, we have developed a simple method to synthesize cubic Iridium nanoparticles with clean surfaces through galvanic reaction. For each synthesis, only one drop of solution containing precursor was employed, which made the synthesis quite economic and efficient. Since the galvanic reaction could be quite fast and the cubic nanoparticles could form in a short time (<30 s) with only one drop of 400 µL solution injecting onto the Cu foil, it made possible the synthesis of cubic Iridium nanoparticles as catalysts at a large scale. This method could also be employed to synthesize other cubic nanoparticles with clean surfaces such as Rh. In the future, we will introduce supports such as graphene to broaden the application of the nanoparticles as synthesized, since the Cu support was not so electrochemically stable and could be corroded at a critical condition, which might be improved by introducing stable supports such as carbon materials.
